# Incidência de Hipertensão Arterial está Associada com Adiposidade em Crianças e Adolescentes

**DOI:** 10.36660/abc.20220070

**Published:** 2023-02-16

**Authors:** Letícia Welser, Karin Allor Pfeiffer, João Francisco de Castro Silveira, Andreia Rosane de Moura Valim, Jane Dagmar Pollo Renner, Cézane Priscila Reuter

**Affiliations:** 1 Universidade de Santa Cruz do Sul Santa Cruz do Sul RS Brasil Universidade de Santa Cruz do Sul, Santa Cruz do Sul, RS – Brasil; 2 Michigan State University East Lansing Michigan EUA Michigan State University, East Lansing, Michigan – EUA; 3 Universidade Federal do Rio Grande do Sul Porto Alegre RS Brasil Universidade Federal do Rio Grande do Sul, Porto Alegre, RS – Brasil

**Keywords:** Doenças Cardiovasculares, Pressão Arterial, Obesidade

## Abstract

**Fundamento:**

O aumento de hipertensão em crianças e adolescentes tem atraído a atenção da comunidade científica, especialmente por sua associação com a epidemia da obesidade.

**Objetivos:**

Descrever a incidência de hipertensão e sua relação com o perfil cardiometabólico e genético em crianças e adolescentes de uma cidade do sul do Brasil em um período de três anos.

**Métodos:**

Este estudo longitudinal acompanhou 469 crianças e adolescentes com idade entre 7 e 17 anos (43,1% do sexo masculino), avaliados em dois momentos. Avaliamos pressão arterial sistólica (PAS), pressão arterial diastólica (PAD), circunferência da cintura (CC), índice de massa corporal (IMC), porcentagem de gordura corporal (%GC), perfil lipídico, glicemia, aptidão cardiorrespiratória (APCR), e polimorfismo rs9939609 (gene *FTO* ) ( *fat mass and obesity* - *associated gene* ). A incidência cumulativa da hipertensão foi calculada, e realizada regressão logística multinominal. A diferença estatística foi estabelecida em p<0,05.

**Resultados:**

Após três anos, a incidência de hipertensão foi de 11,5%. Indivíduos com sobrepeso e indivíduos obesos apresentaram maior probabilidade de se tornarem indivíduos classificados como *borderline* para hipertensão (sobrepeso OR: 3,22; IC95%: 1,08-9,55; obesidade OR: 4,05; IC95%: 1,68-9,75), e indivíduos obesos apresentaram maior probabilidade de se tornarem hipertensos (obesidade OR: 4,84; IC95%: 1,57-14,95). Valores de CC e de %GC considerados de alto risco foram associados com o desenvolvimento de hipertensão (OR: 3,41; IC95%: 126-9,19; OR: 2,49, IC95%: 1,08-5,75, respectivamente).

**Conclusão:**

Encontramos uma incidência de hipertensão em crianças e adolescentes mais alta em comparação a estudos anteriores. Indivíduos com valores mais altos de IMC, CC e %GC no *baseline* apresentaram maior probabilidade de desenvolverem hipertensão, sugerindo a importância da adiposidade no desenvolvimento de hipertensão, mesmo em uma população tão jovem.

## Introdução

O aumento nos níveis pressóricos ao longo do tempo em crianças e adolescentes tem atraído a atenção de profissionais da saúde e da comunidade científica,^1^ especialmente devido à sua associação com a epidemia da obesidade.^2^ De acordo com um estudo recente,^3^ estima-se que a prevalência de hipertensão em crianças e adolescentes no mundo seja de 4%, e a prevalência de pressão arterial elevada, de acordo com as novas diretrizes do *American Academy of Pediatrics* , seja de 15%. Embora essas estimativas de prevalência provavelmente tenham sido subestimadas antes da recente reclassificação,^4^ dados indicam que as taxas nos países em desenvolvimento estejam aumentando.^5^

Nos últimos anos tem sida dada maior atenção à relação entre hipertensão e o desenvolvimento de lesão de órgão-alvo decorrente de disfunção precoce. Os principais fatores de risco modificáveis para prevenir acidente vascular cerebral e outra doenças são hipertensão,^6 , 7^ diabetes mellitus, tabagismo, e dislipidemia, bem como má alimentação/nutrição, sedentarismo e obesidade.^8^ A forte associação entre obesidade e hipertensão conduziu ao desenvolvimento de várias medidas simples, de baixo custo, para avaliação de adiposidade corporal, por exemplo, índice de massa corporal (IMC), circunferência da cintura (CC), e porcentagem de gordura corporal (%GC).^9^ Sabe-se que o risco de hipertensão aumenta com o aumento dos índices de obesidade, elevando as chances de problemas cardiometabólicos.^10^ Está bem documentado na literatura que fatores de risco durante a infância podem levar a complicações cardiorrespiratórias na adolescência^11^ e na fase adulta.^12^

Outro fator de risco importante para hipertensão são os baixos níveis de aptidão cardiorrespiratória (APCR). Dados mostram que níveis mais altos de APCR promovem menor risco de hipertensão.^13^ Variáveis bioquímicas como dislipidemia,^14^ hiperuricemia,^15^ e níveis alterados de glicemia (resistência à insulina)^16^mostraram-se importantes fatores de risco modificáveis para a hipertensão.

Embora a hipertensão tenha sido documentada em vários estudos, há poucos estudos sobre a incidência de hipertensão na população geral.^17^ Estudos abordando a incidência de hipertensão e sua relação com outras variáveis cardiometabólicas, incluindo perfil genético, em crianças e adolescentes são ainda mais escassos. Com base nessas considerações, o objetivo deste estudo foi descrever a incidência de hipertensão, sua relação com variáveis cardiometabólicas e genótipos do polimorfismo rs9939609 do gene FTO em crianças e adolescentes de uma cidade no sul do Brasil.

## Métodos

A população alvo deste estudo de coorte retrospectivo foi crianças e adolescentes voluntários da cidade de Santa Cruz do Sul, RS, Brasil. Todos os procedimentos foram conduzidos em laboratórios, salas e complexo esportivo do campus universitário, e consistiram em avaliação de dados de APCR, dados antropométricos, bioquímicos e de polimorfismo genético.

Os critérios de inclusão para esta subamostra foram estudantes que: a) compareceram às avaliações de 2011 e de 2014; b) participaram da coleta de sangue; c) submeteram-se à avaliação antropométrica completa; d) preencheram todos os formulários; e e) tinham idade entre sete e 17 anos em ambas as avaliações. No total, resultados de 469 estudantes foram analisados, como apresentado na Figura Central .

**Figura Central:**
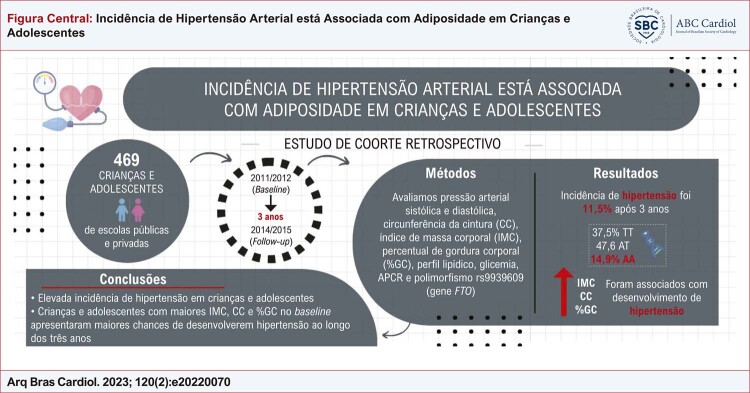
: Incidência de Hipertensão Arterial está Associada com Adiposidade em Crianças e Adolescentes

**Figura 1:**
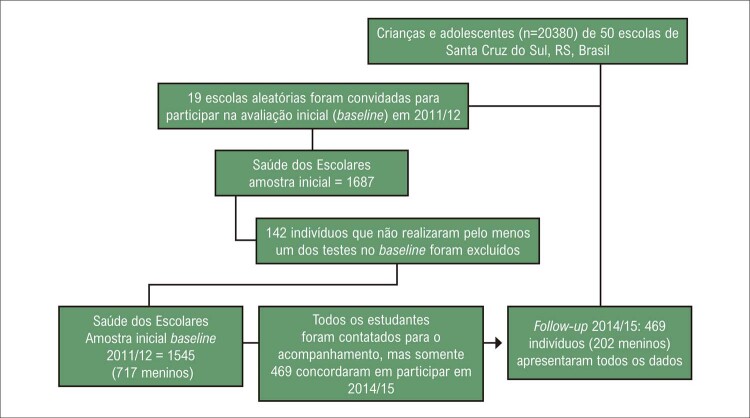
– Fluxograma de seleção da amostra do estudo.

Os participantes deste estudo foram crianças e adolescentes entre sete e 17 anos de idade, de ambos os sexos, de 19 escolas públicas e privadas, estratificados por região (norte, sul, leste, oeste e centro), de áreas urbanas e rurais. A amostra escolhida é oriunda de dois bancos de dados, incluindo somente estudantes que compareceram às avaliações físicas em 2011/2012 ( *baseline* ) e retornaram em 2014/2015 ( *follow-up* ) ( Figura 1 ). Os bancos de dados derivam de um estudo maior chamado “Saúde dos Escolares”, estratificado por grupos. O estudo foi aprovado pelo Comitê de Ética em Pesquisa com Seres Humanos da Universidade de Santa Cruz do Sul - UNISC (números 2959/2011 e 714.216 para o *baseline* e *follow-up* , respectivamente). Todos os pais ou responsáveis foram informados sobre os procedimentos e assinaram um termo de consentimento, autorizando a participação do aluno no estudo.

As variáveis incluídas no estudo foram pressão arterial sistólica (PAS), pressão arterial diastólica (PAD), CC, IMC, %GC, perfil lipídico – triglicerídeos (TG), colesterol total (CT), lipoproteína de alta densidade (HDL-c), lipoproteína de baixa densidade (LDL-c), glicemia, e APCR.

A PAS e a PAD foram medidas duas vezes com o estudante sentado por no mínimo cinco minutos em uma sala quieta. Foram usados um esfigmomanômetro e um estetoscópio no braço direito, e um manguito apropriado para a circunferência do braço. A PAS foi determinada pela detecção do primeiro som de Korotkoff e a PAD pelo som na fase 5 dos sons de Korotkoff, isto é, quando os sons não são mais audíveis ou quando o timbre do som muda. Essas medidas foram classificadas pelos percentis 90 e 95 para *borderline* e hipertensão, de acordo com as Diretrizes Práticas para o Rastreamento e Manejo de Hipertensão em Crianças e Adolescentes.^18^

A medida da CC foi realizada usando uma fita inelástica (Cardiomed®) com 1 mm de precisão. As medidas foram tomadas na região mais estreita do tronco entre as costelas e a crista ilíaca. A CC foi classificada segundo o proposto por Fernández et al.;^19^ a obesidade abdominal foi definida por medidas de CC acima do percentil 75 para sexo e idade. Para o cálculo do IMC, a altura foi medida utilizando um estadiômetro acoplado à balança (Filizola®), a qual foi usada para avaliação do peso corporal, com os participantes descalços, vestindo roupas leves. O IMC foi calculado pela fórmula peso / altura^2^ (kg/m^2^), e os resultados classificados de acordo as curvas de percentis para idade e sexo propostas pela OMS em 2007.^20^ Participantes com valores no percentil <3 foram classificados em baixo peso, no percentil ≥85 como sobrepeso, e no percentil ≥ 97 como obeso. Para a medida da %GC, foram medidas as pregas tricipital e subescapular usando o adipômetro Lange® (MultiMed, Skinfold Caliper, EUA). O cálculo da %GC foi realizado pela equação de Heyward e Stolarczyk^21^ e classificada como muito baixa, baixa, ideal, moderadamente alta, alta, e muito alta de acordo com Lohman.^22^ As três primeiras categorias foram reclassificadas como níveis mais baixos e as demais categorias como níveis mais altos para análises.

Para o perfil lipídico e a glicemia, 10mL de sangue foram coletados da veia braquial após jejum de 12 horas; 5mL foram colocados em tubos Vacutainer® para análise de parâmetros cardiometabólicos, e 5mL em tubos contendo EDTA para outras análises. Níveis de CT, HDL-c, LDL-c, TG e glicemia foram avaliados em amostras do soro. Os valores de CT, HD-c, LDL-c e TG foram classificados de acordo com valores de referência internacionais.^23^ Os participantes foram considerados como dislipidêmicos se apresentassem alteração em pelo menos um desses parâmetros. O LDL-c foi calculado usando a equação de Friedewald.^24^ Os valores de glicemia foram determinados no laboratório de bioquímica do exercício da universidade, utilizando o equipamento automatizado Miura One (I.S.E., Roma, Itália) e o kit comercial DiaSys (DiaSys Diagnostic Systems, Alemanha), e classificados de acordo com os protocolos da *American Diabetes Association* :^25^ normal, pré-diabetes e diabetes. Para as análises estatísticas, as classes de pré-diabetes e diabetes foram agrupadas e consideradas como glicemia elevada.

Para a avaliação do polimorfismo genético rs9939609 do gene associado à massa gorda e obesidade ( *FTO* , do inglês *fat mass and obesity* - *associated gene* ) foi avaliado devido à sua associação (alelo A) com obesidade em estudos prévios no Brasil.^26^ A genotipagem (alelos AA, AT, TT) foi realizada por reação em cadeia da polimerase (PCR) em tempo real utilizando o sistema TaqMan® conforme descrito previamente.^27^

A APCR foi avaliada indiretamente por testes de exercícios submáximos. O teste de caminhada/corrida de nove minutos foi usado no *baseline* , e o teste de seis minutos de caminhada/corrida foi usado no *follow-up* , seguindo-se os protocolos do Projeto Esporte Brasil (PROESP-BR).^28 , 29^ Para ambos os testes, as crianças e os adolescentes foram divididos em grupos, de acordo com a distância do percurso de corrida. Os participantes foram instruídos a correrem a maior distância possível, evitando picos de velocidade intercalados por longas caminhadas. Durante o teste, os participantes foram estimulados verbalmente. Ao final do teste, após um sinal, os estudantes pararam de correr e permaneceram no local em que pararam até que a distância percorrida (em metros) fosse registrada. O resultado foi classificado como uma variável categórica, dicotomizada como valores “altos” e “baixos” de acordo com o manual de testes do PROESP-BR.^30^

### Análise estatística

Os dados foram analisados com o programa *Statistical Package for the Social Sciences* (SPSS), versão 23.0 (IBM, Armonk, NY, EUA). As características da amostra foram descritas nos dois períodos de avaliação (2011/12 e 2014/15) como valores absolutos e porcentagens por sexo, idade, polimorfismo rs9939609 (gene *FTO* ) e cor de pele/etnia. A incidência cumulativa (novos casos de hipertensão) no período de três anos foi calculada.

Um modelo de regressão logística multinomial foi construído, com hipertensão como a variável dependente (normotenso foi a categoria de referência), e IMC, CC, %GC, glicemia, dislipidemia, polimorfismo rs9939609 (gene *FTO* ) e APCR como as variáveis independentes, com *odds ratio* (OR) e intervalo de confiança (IC) de 95% para indicar as chances de mudança de normotenso para *borderline* ou de normotenso para hipertenso. Três modelos foram construídos, uma vez que as medidas de adiposidade estão altamente associadas e não devem ser inseridas em um mesmo modelo como variáveis independentes: Modelo 1 com IMC, Modelo 2 com CC, e Modelo 3 com %GC, todos ajustados para idade, sexo e cor de pele. O nível de significância estatística foi estabelecido em p < 0,05.

## Resultados

Dados dos participantes sobre IMC, CC, %GC, glicemia, dislipidemia, e APCR estão apresentados na Tabela 1 . Das 469 crianças e adolescentes avaliados, 202 (43,1%) eram do sexo masculino, e 77,0% eram brancos. Quanto ao polimorfismo rs9939609 ( *FTO* ), 37,5% foram classificados como alelo TT, 47,6% como AT e 14,9% como AA.

**Tabela 1:** – Descrição do perfil metabólico dos participantes por ano de avaliação

Variáveis	n (%)	
	*Baseline* 2011/12	*Follow-up* 2014/15
**IMC**		
Baixo peso	6 (1,3)	4 (0,9)
Normal	283 (60,3)	289 (61,6)
Sobrepeso	92 (19,6)	90 (19,2)
Obesidade	88 (18,8)	86 (18,3)
**CC**		
Baixo risco	357 (76,1)	366 (78,0)
Alto risco	112 (23,9)	103 (22,0)
**%GC**		
Baixo	268 (57,1)	297 (63,3)
Alto	201 (42,9)	172 (36,7)
**Glicose**		
Normal	356 (83,0)	396 (85,5)
Alto	73 (17,0)	67 (14,5)
**Dislipidemia**		
Não	249 (54,8)	249 (54,8)
Sim	205 (45,2)	205 (45,2)
**APCR**		
Baixo	206 (43,9)	261 (56,5)
Alto	263 (56,1)	201 (43,5)

*IMC: índice de massa corporal; CC: circunferência da cintura; %GC: porcentagem de gordura corporal; APCR: aptidão cardiorrespiratória.*

A Tabela 2 descreve a incidência de pressão arterial alterada ao longo dos três anos. A maioria dos indivíduos continuaram classificados como normotensos, e aproximadamente um terço dos normotensos apresentaram aumento nos níveis sanguíneos (passando para as categorias hipertensão *borderline* /hipertensão). Vale mencionar que um número considerável de indivíduos *borderline* e hipertensos tornaram-se normotensos.

**Tabela 2:** – Comparação longitudinal dos estudantes quanto às categorias de pressão arterial

Pressão arterial	n (%)
Normotenso (mantiveram)	309 (65,9)
Normotenso para *borderline*	60 (12,8)
Normotenso para hipertenso	54 (11,5)
*Borderline* (mantiveram)	2 (0,4)
*Borderline* para normotenso	6 (1,3)
*Borderline* para hipertenso	6 (1,3)
Hipertenso (mantiveram)	9 (1,9)
Hipertenso para normotenso	15 (3,2)
Hipertenso para *borderline*	8 (1,7)

**Figura 2:**
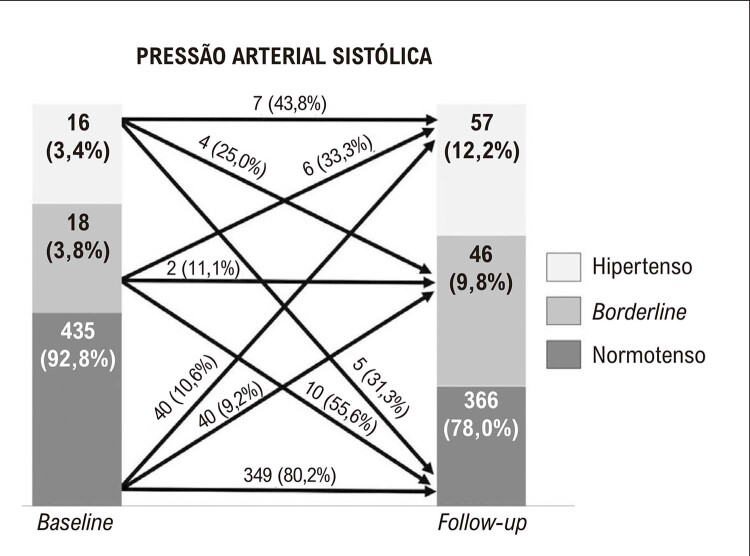
– Incidência de mudanças na pressão arterial sistólica ao longo de três anos.

As Figuras 2 e 3 mostram as mudanças nas classificações da PAS e PAD ao longo do tempo.

**Figura 3:**
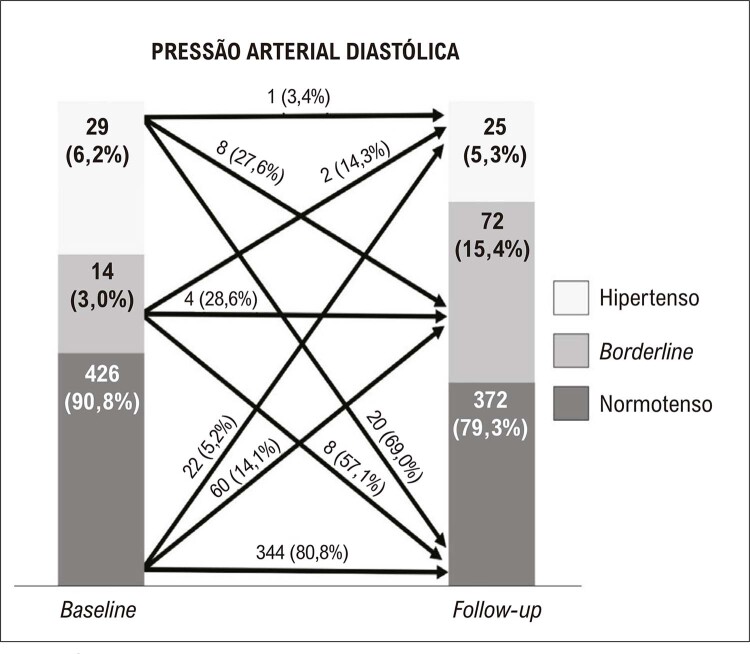
– Incidência de mudanças na pressão arterial diastólica ao longo de três anos.

A Tabela 3 mostra as chances de hipertensão por parâmetros de adiposidade (IMC, CC e %GC) e dados bioquímicos, genéticos e de APCR. Crianças e adolescentes com sobrepeso ou obesidade de acordo com o IMC no *baseline* apresentavam maior probabilidade de se tornarem hipertensos *borderline* ou hipertensos no decorrer de três anos. Valores aumentados de CC foram associados com um risco elevado de se desenvolver hipertensão, e valores maiores de %GC foram associados a mudanças nas categorias de pressão arterial, de normotenso para *borderline* e de normotenso para hipertenso no período do estudo.

**Tabela 3:** – Fatores associados com mudança na pressão arterial durante os três anos de acompanhamento

	Mudança de classificação			
*Baseline*	Normotenso → *Borderline*		Normotenso → Hipertenso	
	OR (IC95%)	p	OR (IC95%)	p
**Modelo 1: IMC**				
IMC				
Baixo peso/Peso normal	1		1	
Sobrepeso	3,22 (1,08-9,55)	0,035	1,69 (0,58-4,90)	0,336
Obesidade	4,05 (1,68-9,75)	0,002	4,84 (1,57-14,95)	0,006
Dislipidemia				
Não	1		1	
Sim	0,86 (0,40-1,84)	0,695	1,60 (0,71-3,61)	0,252
Glicose				
Normal	1		1	
Alto	1,54 (0,60-3,96)	0,370	1,94 (0,74-5,07)	0,177
Polimorfismo rs9939609 ( *FTO* )				
TT	1		1	
AT	1,15 (0,48-2,74)	0,750	0,84 (0,36-1,97)	0,686
AA	1,68 (0,59-4,77)	0,332	0,58 (0,15-2,26)	0,433
APCR				
Alta	1		1	
Baixa	1,60 (0,73-3,51)	0,244	1,19 (0,51-2,81)	0,683
**Modelo 2: CC**				
CC				
Baixo risco	1		1	
Alto risco	2,46 (0,99-6,12)	0,052	3,41 (1,26-9,19)	0,016
Dislipidemia				
Não	1		1	
Sim	0,95 (0,45-2,01)	0,898	1,65 (0,74-3,70)	0,224
Glicose				
Normal	1		1	
Alto	1,78 (0,70-4,49)	0,225	1,92 (0,74-5,01)	0,179
Polimorfismo rs9939609 ( *FTO* )				
TT	1		1	
AT	1,05 (0,45-2,47)	0,902		
AA	1,57 (0,56-4,38)	0,387	0,53 (0,14-2,06)	0,363
APCR				
Alta	1		1	
Baixa	1,65 (0,76-3,59)	0,204	1,34 (0,58-3,09)	0,489
**Modelo 3: %GC**				
%GC				
Baixa	1		1	
Alta	2,28 (1,04-4,97)	0,039	2,49 (1,08-5,75)	0,033
Dislipidemia				
Não	1		1	
Sim	0,93 (0,44-1,96)	0,845	1,58 (0,71-3,52)	0,265
Glicose				
Normal	1		1	
Alta	1,59 (0,63-4,01)	0,327	1,68 (0,65-4,34)	0,280
Polimorfismo rs9939609 ( *FTO* )				
TT	1		1	
AT	1,12 (0,48-2,61)	0,799	0,90 (0,39-2,09)	0,807
AA	1,74 (0,62-4,85)	0,292	0,57 (0,15-2,21)	0,419
APCR				
Alta	1		1	
Baixa	1,61 (0,74-3,49)	0,229	1,35 (0,59-3,08)	0,479

*IMC: índice de massa corporal; CC: circunferência da cintura; %GC: porcentagem de gordura corporal; APCR: aptidão cardiorrespiratória.*

## Discussão

O objetivo deste estudo foi determinar a incidência de hipertensão e relacioná-la às variáveis cardiometabólicas (peso, APCR, e perfil bioquímico) e aos genótipos do polimorfismo rs9939609 do gene FTO de crianças e adolescentes brasileiros Nossos resultados identificaram 12,8% de indivíduos normotensos no *baseline* que se tornaram *borderline* , e 11,5% desses mudaram para hipertensos. Importante mencionar que essas associações se deram independentemente de crescimento ou estágio de desenvolvimento. Além disso, em relação às variáveis de adiposidade, crianças e adolescentes com sobrepeso ou obesidade de acordo com IMC (modelo 1) no *baseline* apresentaram maior probabilidade de se tornarem hipertensos *borderline* , e os obesos apresentaram maior probabilidade de se tornarem hipertensos no decorrer de três anos. Valores de alto risco para CC (modelo 2) foram associados com hipertensão, e valores mais altos de %GC (modelo 3) foram associados com desenvolvimento tanto de hipertensão como de hipertensão *borderline* .

A incidência de níveis elevados de pressão arterial em crianças e adolescentes vem aumentando.^4^ Esse aumento tem sido atribuído à alta incidência de sobrepeso e obesidade nessa população, uma vez que ganho de peso excessivo, principalmente quando associado ao aumento de adiposidade visceral, é uma causa importante de hipertensão.^31^ Segundo um grande estudo com adolescentes brasileiros, a maior prevalência de hipertensão no Brasil encontra-se na região sul, a mesma população avaliada no presente estudo. A região sul do Brasil também apresenta a maior prevalência de obesidade, sobrepeso e sedentarismo.^32^

Nosso estudo mostrou que crianças e adolescentes com sobrepeso ou obesidade de acordo com IMC (modelo 1) no *baseline* apresentaram maior probabilidade de se tornarem *borderline* (OR: 3,22; IC95%: 1,08-9,55; obesidade OR: 4,05; IC95%: 1,68-9,75) ou hipertensos (obesidade OR: 4,84; IC95%: 1,57-14,95) no decorrer de três anos. Ainda, outros estudos com populações de mesma faixa etária mostraram associações entre IMC e níveis elevados de pressão arterial.^33^ Um estudo^34^ similar, porém transversal, teve como objetivo verificar a associação entre sobrepeso/obesidade e pressão arterial elevada em estudantes brasileiros com idade entre seis e 10 anos. A obesidade aumentou em duas vezes a chance de pressão arterial elevada em crianças com idade de 6-7 anos. Em crianças com idade de 8-9 anos, o sobrepeso duplicou o risco de pressão arterial elevada, e a obesidade aumentou em quatro vezes essa chance.^34^ Em outro estudo,^35^ conduzido com crianças e adolescentes chineses com idade entre 7 e 18 anos, uma alta prevalência de pressão arterial elevada foi encontrada em indivíduos com sobrepeso (19%) e obesidade (23,2%).^35^

Em nossos resultados (modelo 2), CC de “alto risco” foi associada ao desenvolvimento de hipertensão no *follow-up* (OR: 3,41; IC95%: 1,26-9,19), o que é corroborado por vários estudos.^36 - 39^ Há achados indicando que tanto a CC como o IMC são bons preditores de níveis elevados de pressão arterial em cada fase da vida,^2^ incluindo um estudo que mostrou que a CC foi um bom preditor de hipertensão, mesmo quando o IMC era normal.^40^ Contudo, outro estudo mostrou que um IMC elevado, mas não CC ou %GC, foi associado com alto risco de hipertensão em crianças chinesas com peso normal.^41^ Como se sabe, a distribuição anormal ou um excesso no tecido adiposo afeta o sistema renina-angiotensina-aldosterona, e o aumento na produção de ácidos graxos não esterificados e de citocinas não inflamatórias (por exemplo, interleucina 6). Ainda, a obesidade está associada com um estado de hiperinsulinemia, que ativa o sistema nervoso simpático. Essas alterações envolvem um aumento na reabsorção renal e no volume intravascular, e vasoconstrição, que resulta em hipertensão arterial.^42^

Uma revisão sistemática sobre adiposidade abdominal e fatores de risco cardiometabólicos mostrou que a medida da pressão arterial foi o parâmetro cardiometabólico mais comum entre os estudos.^43^ A maioria desses estudos confirmou a associação entre níveis elevados de pressão arterial e obesidade abdominal, contudo, a maioria era transversal. Por outro lado, alguns estudos não confirmaram que a CC foi um melhor preditor que o IMC para identificar crianças com níveis elevados de pressão arterial.^44^ Uma meta-análise incluiu 23 estudos observacionais longitudinais e mostrou que a CC e o IMC foram bons preditores de diabetes, mas não de hipertensão.^45^

Ainda, crianças e adolescentes com níveis mais altos de %GC no *baseline* apresentaram maior probabilidade de se tornarem *borderline* (OR: 2,28; IC95%: 1,04-4,97) ou hipertensos (OR: 2,49; IC95%: 1,08-5,75), corroborando alguns achados na literatura.^46 , 47^ Uma porcentagem mais alta de gordura corporal poderia aumentar o risco de hipertensão, e sua redução poderia ser benéfico para a prevenção e controle da hipertensão em crianças, como demonstrado por Tao et al.^47^ Em seu estudo^47^ e no presente estudo, todos os resultados relacionados à adiposidade apontam para a mesma direção – a associação com níveis pressóricos elevados. Um estudo^48^ com uma população adulta mostrou que a perda de peso teve efeitos benéficos sobre a hipertensão e eventos cardiovasculares incidentes. Sabe-se que mudanças no estilo de vida têm um impacto sobre os níveis de pressão arterial e reforçam que a redução de peso é uma importante meta na prevenção primária de eventos cardiovasculares.^48^ Uma redução de 5% no peso corporal está associada a uma diminuição de 20-30% na pressão arterial,^49^ importante indicador de melhora na função vascular.^50^ Outro estudo também mostrou a associação entre níveis de pressão arterial e obesidade, sugerindo que os níveis elevados na pressão possam estar mais relacionados à composição que ao peso corporal, uma vez que, em algumas situações, o peso corporal, e consequentemente o IMC são mais altos, mas a %GC é baixa. Achados do mesmo estudo sugerem que a perda e o ganho de peso, que alteram o perfil antropométrico, têm um grande impacto na reversão e no desenvolvimento de hipertensão, respectivamente.^51^

Um estudo de acompanhamento indicou que a pressão arterial apresenta uma estabilidade moderada da infância à adolescência ou fase inicial adulta, já que as medidas de pressão arterial parecem ser preditores independentes de medidas futuras.^52^ Outro estudo de acompanhamento, desta vez incluindo participantes da fase tardia adulta à fase inicial da adolescência, mostrou estabilidade baixa à moderada para PAS e para PAD ao longo de três anos.^53^ Essa tendência pode ser explicada por mudanças no perfil antropométrico ao longo do tempo,^49^ tais como aumento na adiposidade central, principal fator de risco da síndrome metabólica,^54^ a qual pode levar a mudanças na pressão arterial, nos lipídios circulantes e na glicemia.^50^

No presente estudo, houve casos de pacientes que mudaram do estado de hipertensão *borderline* ou hipertensão para a categoria de normotensos. Uma possível explicação é a influência da escola, onde os alunos participam de atividades físicas que podem melhorar sua condição física e consequentemente sua saúde.

Nenhum dos outros fatores de risco mostrou uma associação estatisticamente significativa com hipertensão *borderline* ou hipertensão em nenhum dos modelos, talvez por se tratar de um estágio muito precoce da vida para promoverem consequências na saúde. Apesar da forte relação entre APCR e composição corporal, do fato de que a APCR tenha se mostrado um importante fator de risco modificável da hipertensão, bem como um forte preditor negativo independente da pressão arterial em crianças e adolescentes,^16^ não encontramos associação estatisticamente significativa entre elas. Assim, a prevenção da doença cardiovascular deveria iniciar-se na infância por meio de um rastreamento regular para hipertensão, aconselhamento para um estilo saudável, e prevenção de fatores de risco modificáveis para IMC, CC e %GC.^55^

O presente estudo tem algumas limitações: nossas análises não controlaram atividade física, estágio de maturação, e comportamentos alimentares; o IMC foi usado como um marcador de adiposidade, embora haja métodos mais diretos para a avaliar. Ainda, a pressão arterial foi medida somente duas vezes por período de avaliação, apesar de recomendações^18^ para três medidas em cada avaliação. Contudo, nosso estudo é comparável à maioria dos estudos epidemiológicos que, por questões de custo e logística, realizam medidas únicas em cada período de avaliação. Como pontos fortes, nosso estudo avaliou uma amostra selecionada aleatoriamente no *baseline* para medir a pressão arterial, pelo método auscultatório, pelo mesmo avaliador em ambos os períodos, e de acordo com as mesmas recomendações.^18^

## Conclusão

O presente estudo longitudinal mostrou um aumento no número de casos, representado pela alta incidência de hipertensão em crianças e adolescentes, em comparação a estudos anteriores. Além disso, nossos resultados mostraram que crianças e adolescentes com medidas maiores de IMC, CC e %GC no *baseline* tinham maior probabilidade de desenvolverem hipertensão no decorrer dos três anos, destacando a importância do cuidado da saúde da criança e do adolescente na prevenção de problemas em idades futuras.

## References

[1] Wang S, Shen G, Jiang S, Xu H, Li M, Wang Z (2017). Nutrient Status of Vitamin D Among Chinese Children. Nutrients.

[2] NCD Risk Factor Collaboration (NCD-RisC) (2017). Worldwide Trends in Body-mass Index, Underweight, Overweight, and Obesity from 1975 to 2016: A Pooled Analysis of 2416 Population-based Measurement Studies in 128·9 Million Children, Adolescents, and Adults. Lancet.

[3] Song P, Zhang Y, Yu J, Zha M, Zhu Y, Rahimi K (2019). Global Prevalence of Hypertension in Children: A Systematic Review and Meta-analysis. JAMA Pediatr.

[4] Sharma AK, Metzger DL, Rodd CJ (2018). Prevalence and Severity of High Blood Pressure Among Children Based on the 2017 American Academy of Pediatrics Guidelines. JAMA Pediatr.

[5] Ibrahim MM (2018). Hypertension in Developing Countries: A Major Challenge for the Future. Curr Hypertens Rep.

[6] Pistoia F, Sacco S, Degan D, Tiseo C, Ornello R, Carolei A (2016). Hypertension and Stroke: Epidemiological Aspects and Clinical Evaluation. High Blood Press Cardiovasc Prev.

[7] Kupferman JC, Zafeiriou DI, Lande MB, Kirkham FJ, Pavlakis SG (2017). Stroke and Hypertension in Children and Adolescents. J Child Neurol.

[8] Guzik A, Bushnell C (2017). Stroke Epidemiology and Risk Factor Management. Continuum (Minneap Minn).

[9] Ding W, Cheng H, Chen F, Yan Y, Zhang M, Zhao X (2018). Adipokines are Associated With Hypertension in Metabolically Healthy Obese (MHO) Children and Adolescents: A Prospective Population-Based Cohort Study. J Epidemiol.

[10] Jayedi A, Rashidy-Pour A, Khorshidi M, Shab-Bidar S (2018). Body Mass Index, Abdominal Adiposity, Weight Gain and Risk of Developing Hypertension: A Systematic Review and Dose-response Meta-analysis of More Than 2.3 Million Participants. Obes Rev.

[11] Redwine KM, Acosta AA, Poffenbarger T, Portman RJ, Samuels J (2012). Development of Hypertension in Adolescents with Pre-hypertension. J Pediatr.

[12] Barrington DS, James SA (2017). Receipt of Public Assistance During Childhood and Hypertension Risk in Adulthood. Ann Epidemiol.

[13] Sui X, Sarzynski MA, Lee DC, Lavie CJ, Zhang J, Kokkinos PF (2017). Longitudinal Patterns of Cardiorespiratory Fitness Predict the Development of Hypertension Among Men and Women. Am J Med.

[14] Genovesi S, Giussani M, Orlando A, Battaglino MG, Nava E, Parati G (2019). Prevention of Cardiovascular Diseases in Children and Adolescents. High Blood Press Cardiovasc Prev.

[15] Viazzi F, Antolini L, Giussani M, Brambilla P, Galbiati S, Mastriani S (2013). Serum Uric acid and Blood Pressure in Children at Cardiovascular Risk. Pediatrics.

[16] Nathan BM, Moran A (2008). Metabolic Complications of Obesity in Childhood and Adolescence: More than Just Diabetes. Curr Opin Endocrinol Diabetes Obes.

[17] Lacruz ME, Kluttig A, Hartwig S, Löer M, Tiller D, Greiser KH (2015). Prevalence and Incidence of Hypertension in the General Adult Population: Results of the CARLA-Cohort Study. Medicine (Baltimore).

[18] Flynn JT, Kaelber DC, Baker-Smith CM, Blowey D, Carroll AE, Daniels SR (2017). Clinical Practice Guideline for Screening and Management of High Blood Pressure in Children and Adolescents. Pediatrics.

[19] Fernández JR, Redden DT, Pietrobelli A, Allison DB (2004). Waist Circumference Percentiles in Nationally Representative Samples of African-American, European-American, and Mexican-American Children and Adolescents. J Pediatr.

[20] World Health Organization (2007). Child Growth Reference Data for 5-19 Years Standards.

[21] Heyward V, Stolarczyk L (2000). Avaliação da Composição Corporal Aplicada.

[22] Lohman TG (1987). The Use of Skinfold to Estimate Body Fatness on Children and Youth. J Phys Ed.

[23] Expert Panel on Integrated Guidelines for Cardiovascular Health and Risk Reduction in Children and Adolescents, National Heart, Lung, and Blood Institute (2011). Expert Panel on Integrated Guidelines for Cardiovascular Health and Risk Reduction in Children and Adolescents: Summary Report. Pediatrics.

[24] Friedewald WT, Levy RI, Fredrickson DS (1972). Estimation of the Concentration of Low-density Lipoprotein Cholesterol in Plasma, Without Use of the Preparative Ultracentrifuge. Clinical Chemistry.

[25] American Diabetes Association (2018). Classification and Diagnosis of Diabetes: Standards of Medical Care in Diabetes-2018. Diabetes Care.

[26] Todendi PF, Klinger EI, Geraldo ACR, Brixner L, Reuter CP, Lindenau JDR (2019). Genetic Risk Score Based on Fat Mass and Obesity-associated, Transmembrane Protein 18 and Fibronectin Type III Domain Containing 5 Polymorphisms is Associated with Anthropometric Characteristics in South Brazilian Children and Adolescents. Br J Nutr.

[27] Reuter CP, Burgos MS, Bernhard JC, Tornquist D, Klinger EI, Borges TS (2016). Association Between Overweight and Obesity in Schoolchildren with rs9939609 Polymorphism (FTO) and Family History for Obesity. J Pediatr (Rio J).

[28] Projeto Esporte Brasil (2009). Manual de Aplicação De Medidas e Testes, Normas e Critérios de Avaliação.

[29] Projeto Esporte Brasil (2012). Manual de Aplicação de Medidas e Testes, Normas e Critérios de Avaliação.

[30] Gaya AR, Gaya A, Pedretti A, Mello J (2021). Projeto Esporte Brasil: Manual de Medidas, Testes e Avaliações.

[31] Wühl E (2019). Hypertension in Childhood Obesity. Acta Paediatr.

[32] Bloch KV, Klein CH, Szklo M, Kuschnir MC, Abreu GA, Barufaldi LA (2016). ERICA: Prevalences of Hypertension and Obesity in Brazilian Adolescents. Rev Saude Publica.

[33] Zhao Y, Wang L, Xue H, Wang H, Wang Y (2017). Fast Food Consumption and its Associations with Obesity and Hypertension Among Children: Results from the Baseline Data of the Childhood Obesity Study in China Mega-cities. BMC Public Health.

[34] Pereira FEF, Teixeira FDC, Kac G, Soares EA, Ribeiro BG (2020). Overweight and Obesity Associated with High Blood Pressure: A Cross-sectional Study in Brazilian Students. Rev Esc Enferm USP.

[35] Zhang CX, Shi JD, Huang HY, Feng LM, Ma J (2012). Nutritional Status and its Relationship with Blood Pressure Among Children and Adolescents in South China. Eur J Pediatr.

[36] Cruz NRC, Cardoso PC, Frossard TNSV, Ferreira FO, Brener S, Gomides AFF (2019). Waist Circumference as High Blood Pressure Predictor in School Age Children. Cien Saude Colet.

[37] Andrade GN, Matoso LF, Miranda JWB, Lima TF, Gazzinelli A, Vieira EW (2019). Anthropometric Indicators Associated with High Blood Pressure in Children Living in Urban and Rural Areas. Rev Lat Am Enfermagem.

[38] Dong B, Wang Z, Yang Y, Wang HJ, Ma J (2016). Intensified Association Between Waist Circumference and Hypertension in Abdominally Overweight Children. Obes Res Clin Pract.

[39] Christofaro DGD, Farah BQ, Vanderlei LCM, Delfino LD, Tebar WR, Barros MVG (2018). Analysis of Different Anthropometric Indicators in the Detection of High Blood Pressure in School Adolescents: A Cross-sectional Study with 8295 Adolescents. Braz J Phys Ther.

[40] Pazin DC, Rosaneli CF, Olandoski M, Oliveira ERN, Baena CP, Figueredo AS (2017). Waist Circumference is Associated with Blood Pressure in Children with Normal Body Mass Index: A Cross-Sectional Analysis of 3,417 School Children. Arq Bras Cardiol.

[41] Xu RY, Zhou YQ, Zhang XM, Wan YP, Gao X (2018). Body Mass Index, Waist Circumference, Body Fat Mass, and Risk of Developing Hypertension in Normal-weight Children and Adolescents. Nutr Metab Cardiovasc Dis.

[42] Bogaert YE, Linas S (2009). The Role of Obesity in the Pathogenesis of Hypertension. Nat Clin Pract Nephrol.

[43] Kelishadi R, Mirmoghtadaee P, Najafi H, Keikha M (2015). Systematic Review on the Association of Abdominal Obesity in Children and Adolescents with Cardio-metabolic Risk Factors. J Res Med Sci.

[44] Ma C, Wang R, Liu Y, Lu Q, Lu N, Tian Y (2016). Performance of Obesity Indices for Screening Elevated Blood Pressure in Pediatric Population: Systematic Review and Meta-analysis. Medicine (Baltimore).

[45] Seo DC, Choe S, Torabi MR (2017). Is Waist Circumference ≥102/88cm Better Than Body Mass Index ≥30 to Predict Hypertension and Diabetes Development Regardless of Gender, Age Group, and Race/Ethnicity? Meta-analysis. Prev Med.

[46] Qiong W (2008). Analysis of the relationship between body fat content and blood pressure of high school students in Wuhan. Chinese Health at School.

[47] Tao RW, Wan YH, Zhang H, Wang YF, Wang B, Xu L (2016). Relationship between hypertension and percentage of body fat, in children of Anhui province.

[48] Markus MR, Ittermann T, Baumeister SE, Troitzsch P, Schipf S, Lorbeer R (2015). Long-term Changes in Body Weight are Associated with Changes in Blood Pressure Levels. Nutr Metab Cardiovasc Dis.

[49] Sociedade Brasileira de Cardiologia (2016). VII Diretriz Brasileira de Hipertensão Arterial. Arq Bras Cardiol.

[50] Polito LFT, Zanetti MC, Sanches IC, Montenegro CGSP, Brandão MRF, Junior AJF (2018). Obesidade e seus Fatores Associados. Propostas para Promoção da Saúde a partir do Exercício Físico e da Aderência a ele Associada.

[51] Ittermann T, Werner N, Lieb W, Merz B, Nöthlings U, Kluttig A (2019). Changes in Fat Mass and Fat-free-mass are Associated with Incident Hypertension in Four Population-based Studies from Germany. Int J Cardiol.

[52] Sarganas G, Schaffrath Rosario A, Niessner C, Woll A, Neuhauser HK (2018). Tracking of Blood Pressure in Children and Adolescents in Germany in the Context of Risk Factors for Hypertension. Int J Hypertens.

[53] Silveira JF, Reuter CP, Welser L, Pfeiffer KA, Andersen LB, Pohl HH (2021). Tracking of Cardiometabolic Risk in a Brazilian Schoolchildren Cohort: A 3-year Longitudinal Study. J Sports Med Phys Fitness.

[54] International Diabetes Federation (2015). IDF Diabetes Atlas.

[55] Kapur G, Mattoo TK (2018). Pediatric Hypertension.

