# Supplementary material for: Incidência de Hipertensão Arterial está Associada com Adiposidade em Crianças e Adolescentes
Source: Arq Bras Cardiol. 2023 Feb 16;120(2):e20220070. [Article in Portuguese] doi: 10.36660/abc.20220070 (PMC12080599; doi:10.36660/abc.20220070)
Supplement: Translation

# Translation: Incidence of Arterial Hypertension is Associated with Adiposity in Children and Adolescents

**Authors:** Letícia Welser, Karin Allor Pfeiffer, João Francisco de Castro Silveira, Andreia Rosane de Moura Valim, Jane Dagmar Pollo Renner, Cézane Priscila Reuter

**Affiliations:** 1 Brazil Universidade de Santa Cruz do Sul, Santa Cruz do Sul, RS – Brazil; 2 USA Michigan State University, East Lansing, Michigan – USA; 3 Brazil Universidade Federal do Rio Grande do Sul, Porto Alegre, RS – Brazil

**Keywords:** Cardiovascular Diseases, Arterial Pressure, Obesity

## Abstract

**Background:**

The increase of hypertension in children and adolescents has attracted the attention of the scientific community largely due to its association with the obesity epidemic.

**Objectives:**

To describe the incidence of hypertension and its relationship with the cardiometabolic and genetic profile in children and adolescents from a city in southern Brazil in a three-year period.

**Methods:**

This longitudinal study followed 469 children and adolescents, aged 7-17 years old (43.1% boys), assessed at two-time points. We evaluated systolic and diastolic blood pressures (SBP and DBP), waist circumference (WC), body mass index (BMI), body fat percentage (%BF), lipid profile, glucose, cardiorespiratory fitness (CRF), and rs9939609 Polymorphism ( *FTO* ). Cumulative incidence of hypertension was calculated, and multinomial logistic regression was conducted. The statistical significance was established as p < 0.05.

**Results:**

After three years, the incidence of hypertension was 11.5%. Overweight or obese individuals were more likely to become borderline hypertensive (overweight OR: 3.22, 95% CI: 1.08-9.55; obesity OR: 4.05, 95% CI: 1.68-9.75), and obese individuals were more likely to become hypertensive (obesity OR: 4.84, 95% CI: 1.57-14.95). High-risk WC and %BF values were associated with hypertension development (OR: 3.41, 95% CI: 1.26-9.19; OR: 2.49, 95% CI: 1.08-5.75, respectively).

**Conclusions:**

We found a higher incidence of hypertension in children and adolescents as compared with previous studies. Individuals with higher values of BMI, WC and %BF at baseline were more likely to develop hypertension, suggesting the importance of adiposity in the development of hypertension even in such a young population.

## Introduction

The increase over time in blood pressure levels in children and adolescents has attracted the attention of health professionals and the scientific community,^1^largely due to its association with the obesity epidemic.^2^According to a recent study,^3^it is estimated that the worldwide prevalence of hypertension is 4% in children and adolescents, and the prevalence of high blood pressure according to the new guidelines of the American Academy of Pediatrics is 15%. Although these prevalence estimates may have been underestimated before the recent reclassification,^4^data have indicated that the rates in developing countries are on the rise.^5^

Over the last few years more attention has been given to the relationship between hypertension and the development of end-organ damage emanating from early dysfunctions. The main modifiable risk factors to prevent strokes and other health issues are hypertension,^6 , 7^diabetes mellitus, tobacco smoking, and hyperlipidemia, as well as poor diet/nutrition, physical inactivity, and obesity.^8^The strong association between obesity and hypertension prompted the development of several simple and low-cost measures to assess body adiposity, e.g., body mass index (BMI), waist circumference (WC) and body fat percentage (BF).^9^It is known that the risk of hypertension increases with the increase in obesity indexes, increasing the chances of cardiometabolic problems.^10^It is well documented in the literature that early risk factors during childhood can lead to cardiorespiratory complications in adolescence^11^and adulthood.^12^

Another important risk factor for hypertension is low levels of cardiorespiratory fitness (CRF). Data show that higher levels of CRF provide lower risk of hypertension.^13^Biochemical variables such as dyslipidemia,^14^hyperuricemia,^15^and altered levels of serum glucose (i.e., insulin resistance)^16^have shown to be important modifiable risk factors for hypertension, but CRF has an independent, predictive, and powerful negative association with blood pressure among children and adolescents.

Although the prevalence of hypertension has been well documented in several studies, there are few studies regarding incidence of hypertension in the general population.^17^Studies addressing the incidence of high blood pressure and its relationship with other cardiometabolic variables, including genetic profile, in children and adolescents are even scarcer. Based on these considerations, the aim of this study is to describe the incidence of hypertension and its relationship with cardiometabolic (weight status, CRF and biochemical profile) variables and genotypes of the rs9939609 polymorphism of the FTO gene in Brazilian children and adolescents.

## Methods

The target population of this retrospective cohort research were volunteer children and adolescents of the city of Santa Cruz do Sul, in Rio Grande do Sul state, Brazil. All procedures were conducted at laboratories, rooms, and sports complex of the university campus, and consisted of assessment of CRF, anthropometry, laboratory data, and genetic polymorphism.

Inclusion criteria for this subsample were students who: a) attended the 2011 assessment and returned in 2014; b) took part in the blood collection; c) underwent complete anthropometric assessment; d) filled out all the forms; and e) were in the range between seven and 17 years old in both assessments. In total, results of 469 students were analyzed, as are shown in Central Figure .

**Central Illustration:** : Incidence of Arterial Hypertension is Associated with Adiposity in Children and Adolescents

The participants of this study were children and adolescents between 7-17 years old, of both sexes, from 19 public and private schools, stratified by regions (north, south, east, west and center), from urban and rural areas. The sample selected is from two databases, including only the students who attended the physical assessments in 2011/2012 (baseline) and returned in 2014/2015 (follow-up) ( Figure 1 ). The databases come from a larger study named “Schoolchildren’s Health Study”, stratified by clusters. This research was approved by the Committee of Ethics in Research with Human Subjects of the University of Santa Cruz do Sul – UNISC (approval numbers 2959/2011 and 714.216 for baseline and follow-up, respectively). All parents or guardians were informed about the procedures and signed an informed consent form, authorizing the participation of the student in the study.

**Figure 1:** – Flowchart of study sample selection.

The variables included in the present study were systolic blood pressure (SBP), diastolic blood pressure (DBP), WC, BMI, %BF, lipid profile (triglyceride [TG], total cholesterol [TC], high-density lipoprotein cholesterol [HDL-C], low-density lipoprotein cholesterol [LDL-C]), glucose, and CRF.

SBP and DBP were measured twice with the student seated for at least five minutes in a quiet room. A sphygmomanometer and stethoscope were used on the right arm with an appropriate cuff for the arm circumference. SBP was determined by the first Korotkoff sound and DBP by the fifth Korotkoff sound, that is, when the sounds are no longer audible or when the timbre of the sound changes. These measurements were classified by the 90th and 95th percentiles for borderline and hypertension according to the Clinical Practice Guideline for Screening and Management of High Blood Pressure in Children and Adolescents.^18^

WC was measured to the nearest 1mm using a non-elastic tape (Cardiomed®). Measurement was taken at the narrowest part of the trunk between the ribs and the iliac crest. WC was classified as proposed by Fernández et al.;^19^abdominal obesity was defined as measures above the 75th percentile, according to sex and age. To calculate BMI, height was measured using a stadiometer coupled to the anthropometric scale (Filizola®), which was used for weight assessment, measured for each participant in light clothing without shoes. The formula BMI = weight / height^2^(kg/m^2^) was used. The result was classified according to the WHO (2007)^20^percentile curves for age and sex. Participants with percentile <3 were classified as underweight, percentile ≥85 as overweight and percentile ≥ 97 as obese. To measure %BF, tricipital and subscapular skinfolds were measured using the Lange® caliper (MultiMed, Skinfold Caliper, USA). The %BF calculation was performed using the Heyward and Stolarczyk^21^equation and classified as very low; low; ideal; moderately high; high; and very high according to Lohman.^22^The first three categories were reclassified as lower levels and the other categories as higher levels for analyses.

For the lipid profile and glucose, 10mL of blood were collected from the brachial vein after a 12-hour fast; 5mL were placed in dry Vacutainer® tubes for analysis of cardiometabolic parameters, and 5mL were placed in the EDTA Vacutainer® tubes for further analysis. Levels of TC, HDL-C, LDL-C, TG, and glucose were evaluated in serum samples. The TC, HDL-C, LDL-C, and TG values were classified according to international reference values.^23^Participants were considered dyslipidemic if at least one of the parameters listed above was found to be altered. LDL-C was calculated using the Friedewald formula.^24^Blood glucose levels were determined at the Exercise Biochemistry Laboratory of the university using the automated equipment Miura One (I.S.E., Rome, Italy) and a DiaSys (DiaSys Diagnostic Systems, Germany) commercial kit, and classified according to the American Diabetes Association protocols:^25^normal, pre-diabetes and diabetes. For statistical purposes, the pre-diabetes and diabetes classes were grouped and considered as “high” glucose.

For genetic polymorphism evaluation, the *rs9939609 FTO* (fat mass and obesity-associated gene) was assessed due to its association (A allele) with the presence of obesity in previous studies in Brazil.^26^Genotyping (alleles AA, AT, TT) was performed by real-time polymerase chain reaction (PCR), using TaqMan™ probes as previously described.^27^The Hardy-Weinberg equilibrium was tested and confirmed (p>0.05).

CRF was assessed by indirect submaximal exercise tests. The 9-minute walk/run test was used at baseline, and the 6-minute walk/run test was used in the follow-up, according to the protocols of the Projeto Esporte Brasil (PROESP-BR).^28 , 29^For both tests the children and adolescents were divided in groups that were adequate for the running track dimensions. They were instructed to run as long as possible, avoiding velocity peaks interspersed by long walks. During the test, the participants were verbally encouraged. At the end of the test, after a signal was given, the students stopped running and remained in place where they were standing until the distance traveled (in meters) was recorded. The result given in meters was classified as a categorical variable, dichotomized into “higher” and “lower” values according to the PROESP-BR test manual.^30^

### Statistical analysis

The data were analyzed with Statistical Package for the Social Sciences (SPSS) software, version 23.0 (IBM, Armonk, NY, USA). The characteristics of the sample were described in the two assessed periods, 2011/12 (baseline) and 2014/15 (follow-up), as absolute and percentage values by sex, age, rs9939609 Polymorphism ( *FTO* )and skin color/ethnicity. The cumulative incidence (new cases of hypertension) over three years was calculated.

A multinomial logistic regression model was constructed, with hypertension as the dependent variable (normotensive was the reference category) and BMI, WC, %BF, glucose, dyslipidemia, rs9939609 Polymorphism ( *FTO* ) and CRF as the independent variables, with odds ratio (OR) and 95% confidence interval (CI) to indicate the odds of changing from normotensive to borderline or from normotensive to hypertensive. Three models were constructed, since adiposity measures are highly associated and should not be placed in the same model as independent variables: Model 1 with BMI, Model 2 with WC, and Model 3 with %BF, all adjusted for age, sex, and skin color. The level of statistical significance was established as p < 0.05.

## Results

Participants’ characteristics of BMI, WC, %BF, glucose, dyslipidemia, and CRF are shown in Table 1 . Of the 469 children and adolescents evaluated, 202 (43.1%) were male, and 77.0% were white. Regarding the rs9939609 Polymorphism *(FTO)* 37.5% were classified as allele TT, 47.6% as AT and 14.9% as AA.

**Table 1:** – Description of the participants’ metabolic profile by year of assessment

Variables	n (%) Baseline	Follow-up
**BMI**		
Underweight	6 (1.3)	4 (0.9)
Normal	283 (60.3)	289 (61.6)
Overweight	92 (19.6)	90 (19.2)
Obesity	88 (18.8)	86 (18.3)
**WC**		
Lower risk	357 (76.1)	366 (78.0)
Higher risk	112 (23.9)	103 (22.0)
**%BF**		
Lower	268 (57.1)	297 (63.3)
Higher	201 (42.9)	172 (36.7)
**Glucose**		
Normal	356 (83.0)	396 (85.5)
High	73 (17.0)	67 (14.5)
**Dyslipidemia**		
No	249 (54.8)	249 (54.8)
Yes	205 (45.2)	205 (45.2)
**CRF**		
Lower	206 (43.9)	261 (56.5)
Higher	263 (56.1)	201 (43.5)

*BMI: body mass index; WC: waist circumference; %BF: percentage of body fat; CRF: cardiorespiratory fitness.*

Table 2 describes the incidence of altered blood pressure over three years. Most subjects remained classified as normotensive, while approximately one third of the normotensive sample had increases in their blood pressure levels (moving to the borderline/hypertensive categories). It should be noted that a fair number of borderline and hypertensive individuals became normotensive.

**Table 2:** – Longitudinal comparison of participants according to blood pressure categories

Blood Pressure	n (%)
Normotensive (maintaining)	309 (65.9)
Normotensive to borderline	60 (12.8)
Normotensive to hypertensive	54 (11.5)
Borderline (maintaining)	2 (0.4)
Borderline to normotensive	6 (1.3)
Borderline to hypertensive	6 (1.3)
Hypertensive (maintaining)	9 (1.9)
Hypertensive to normotensive	15 (3.2)
Hypertensive to borderline	8 (1.7)

Figures 2 and 3 show the changes in ratings of PAS and PAD over time.

**Figure 2:** – Changes in the incidence of systolic blood pressure over three years.

**Figure 3:** – Changes in the incidence of diastolic blood pressure over three years.

Table 3 shows the odds of hypertension by adiposity parameters (BMI, WC, and %BF) and biochemical data, genotype, and CRF level. Children and adolescents who were overweight and obese according to baseline BMI were more likely to become borderline or hypertensive over three years. Increased WC values were associated with a high risk for hypertension, and increased %BF was associated with changes in blood pressure category, from normotensive to borderline, and from normotensive to hypertensive over the study period.

**Table 3:** – Factors associated with changes of blood pressure classification over three years of follow-up

	Blood pressure modification across 3 years			
Baseline	Normotensive Borderline		Normotensive Hypertensive	
	OR (95% CI)	p	OR (95% CI)	p
**Model 1: BMI**				
BMI				
Underweight/Normal weight	1		1	
Overweight	3.22 (1.08-9.55)	0.035	1.69 (0.58-4.90)	0.336
Obesity	4.05 (1.68-9.75)	0.002	4.84 (1.57-14.95)	0.006
Dyslipidemia				
No	1		1	
Yes	0.86 (0.40-1.84)	0.695	1.60 (0.714-3.61)	0.252
Glucose				
Normal	1		1	
High	1.54 (0.60-3.96)	0.370	1.94 (0.74-5.07)	0.177
*FTO* rs9939609				
TT	1		1	
AT	1.15 (0.482.74)	0.750	0.84 (0.36-1.97)	0.686
AA	1.68 (0.59-4.77)	0.332	0.58 (0.15-2.26)	0.433
CRF				
Higher	1		1	
Lower	1.60 (0.73-3.51)	0.244	1.19 (0.51-2.81)	0.683
**Model 2: WC**				
WC				
Low risk	1		1	
High risk	2.46 (0.99-6.12)	0.052	3.41 (1.26-9.19)	0.016
Dyslipidemia				
No	1		1	
Yes	0.95 (0.45-2.01)	0.898	1.65 (0.74-3.70)	0.224
Glucose				
Normal	1		1	
High	1.78 (0.70-4.49)	0.225	1.92 (0.74-5.01)	0.179
*FTO* rs9939609				
TT	1		1	
AT	1.05 (0.45-2.47)	0.902		
AA	1.57 (0.56-4.38)	0.387	0.53 (0.14-2.06)	0.363
CRF				
Higher	1		1	
Lower	1.65 (0.76-3.59)	0.204	1.34 (0.58-3.09)	0.489
**Model 3: %BF**				
%BF				
Lower	1		1	
Higher	2.28 (1.04-4.97)	0.039	2.49 (1.08-5.75)	0.033
Dyslipidemia				
No	1		1	
Yes	0.93 (0.44-1.96)	0.845	1.58 (0.71-3.52)	0.265
Glucose				
Normal	1		1	
High	1.59 (0.63-4.01)	0.327	1.68 (0.65-4.34)	0.280
*FTO* rs9939609				
TT	1		1	
AT	1.12 (0.48-2.61)	0.799	0.90 (0.39-2.09)	0.807
AA	1.74 (0.62-4.85)	0.292	0.57 (0.15-2.21)	0.419
CRF				
Higher	1		1	
Lower	1.61 (0.74-3.49)	0.229	1.35 (0.59-3.08)	0.479

*BMI: body mass index; WC: waist circumference; %BF: percentual of body fat; CRF: cardiorespiratory fitness*

## Discussion

The aim of this study was to determine the incidence of hypertension and to relate it with cardiometabolic variables (weight status, CRF, and biochemical profile) and rs9939609 Polymorphism ( *FTO* ) of Brazilian children and adolescents. Our results identified 12.8% of normotensive subjects at baseline who became borderline, and 11.5% of them changed to hypertensive. Importantly, all these associations were independent of growth and development. Furthermore, regarding the adiposity variables, children and adolescents who were overweight or obese according to BMI (model 1) at baseline were more likely to become borderline, while those who were obese were more likely to become hypertensive over three years. High-risk values for WC (model 2) were associated with hypertension, and higher %BF values (model 3) were associated with the development of both borderline hypertension and hypertension.

The incidence of high blood pressure levels has been rising in children and adolescents.^4^This increase is believed to be due to the high incidence of overweight and obesity in this population, since excess weight gain, especially when associated with increased visceral adiposity, is a major cause of hypertension.^31^According to a large study including Brazilian adolescents, the highest prevalence of hypertension in Brazil are in the south region, the same population assessed in the present study. The south region of Brazil also has the highest prevalence of overweight and obesity and the most physically inactive population.^32^

Our study demonstrated that children and adolescents who were overweight and obese according to BMI (model 1) at baseline were more likely to become borderline (overweight OR: 3.22, 95% CI: 1.08-9.55; obesity OR: 4.05, 95% CI: 1.68-9.75) or hypertensive (obesity OR: 4.84, 95% CI: 1.57-14.95) over three years. In addition, other studies with same-age populations have shown associations between BMI and elevated rates of blood pressure.^33^A similar but cross-sectional study^34^aimed to verify the association between overweight/obesity and high blood pressure in Brazilian students aged between six and 10 years old. Obesity increased by twice the chance of high blood pressure among children aged 6-7 years old. In children aged 8-9 years old, overweight doubled the chance of high blood pressure, while obesity quadrupled that chance.^34^In another study,^35^conducted with Chinese children and adolescents aged between 7 and 18 years old, a high prevalence of high blood pressure was also found among those who had overweight (19%) and obesity (23.2%).^35^

In our results (model 2), “high-risk” WC classification was associated with the development of hypertension at follow-up (OR: 3.41, 95% CI: 1.26-9.19), which is supported by several studies.^36 - 39^There are findings that both WC and BMI were good predictors of elevated levels of blood pressure in every phase of life,^2^including one study that showed that WC was a good predictor of hypertension, even when the BMI was normal.^40^Another study, however, showed that high BMI, but not WC or %BF, was associated with high risk of hypertension in normal-weight Chinese children.^41^As it is known, abnormal distribution or excess of fat tissue impacts the renin-angiotensin-aldosterone system and the rise in production of non-esterified fatty acids and inflammatory cytokines (e.g., interleukin 6). Also, obesity is associated with a hyperinsulinemic state, which activates the sympathetic nervous system. These changes involve an increase in renal sodium reabsorption and intravascular volume, and vasoconstriction, which ultimately result in arterial hypertension.^42^

A systematic review of abdominal adiposity and cardiometabolic risk factors showed that blood pressure measurement was the most common cardiometabolic parameter among the studies.^43^Importantly, most of these studies confirmed the association between elevated levels of blood pressure and abdominal obesity; however, most were cross-sectional. On the other hand, some studies did not support WC as a better predictor than BMI to identify children with elevated levels of blood pressure.^44^A metanalysis included 23 longitudinal observation studies and found that WC and BMI were good predictors of diabetes but not hypertension.^45^

Also, children and adolescents with higher levels of %BF at baseline were more likely of becoming borderline (OR: 2.28, 95% CI: 1.04-4.97) or hypertensive (OR: 2.49, 95% CI: 1.08-5.75), corroborating some findings in the literature.^46 , 47^A higher percentage of body fat could increase the risk of hypertension, and its reduction would be beneficial for the prevention and control of hypertension in children as shown by Tao et al.^47^In their study^47^and in the current study, all results related to adiposity point in the same direction: an association with high blood pressure. A study^48^in an adult population showed that weight loss had beneficial effects on incident hypertension and cardiovascular events. It is recognized that lifestyle changes have an impact on blood pressure levels and reinforce that weight reduction is an important goal for the primary prevention of cardiovascular events.^48^A 5% reduction of total body weight is associated with decrease of 20-30% in blood pressure levels^49^which is an important index of vascular function improvement.^50^Also, another study supported the association between blood pressure levels and obesity suggesting that high blood pressure might be more related to body composition rather than body weight itself, since in some situations body weight, and consequently BMI is high, but the %BF is low. The findings of the same study still suggest that losing or gaining weight, which changes the anthropometric profile, has a substantial impact on the reversal or development of hypertension, respectively.^51^

A tracking study indicated that blood pressure shows a moderate stability from childhood into adolescence or early adulthood, since blood pressure measurements seem to be a strong independent predictor for future measurements.^52^Another tracking study, this time including participants from late childhood to early adolescence, showed low-to-moderate stability to SBP and DBP over three years.^53^This trend may be explained by changes in the anthropometric profile over time^49^such as increase of central adiposity, the main risk factor for metabolic syndrome,^54^which can trigger changes alterations in blood pressure, blood lipids and glucose.^50^In the current study, there were also cases of patients who that changed from borderline or hypertension down to the normotensive category. One possible explanation is the influence of school, in which individuals participate in physical activities that can improve their physical status and consequently their health.

None of the other risk factors showed a statistically significant association with borderline or hypertension in any of the models, perhaps because it is too early in life to show some consequence in health. Even though CRF and body composition are highly related, and CRF has shown to be an important modifiable risk factor for hypertension, and independent and powerful negative predictor of blood pressure among children and adolescents,^16^we found no statistically significant association between them. Therefore, the prevention of cardiovascular disease should begin during childhood through regular screening for hypertension, counseling for a healthy lifestyle, and prevention of modifiable risk factors as BMI, WC, and %BF.^55^

The present study has some limitations: our analyses did not control for physical activity, maturation stage, and dietary behaviors; BMI was used as an adiposity marker, even though there are more direct methods for assessing adiposity. Also, blood pressure was measured only twice in each assessment period, despite recommendations^18^for three measurements at each assessment. However, our study was comparable to most epidemiological studies which, for logistical and cost reasons, take single measurements at each assessment period. As strengths, this longitudinal study used a randomly selected sample at baseline to measure blood pressure, using the auscultatory method, by the same evaluator in both periods and according to the same recommendations.^18^

## Conclusion

This longitudinal study showed that there is an increase in the number of new cases, represented by a high incidence of hypertension in children and adolescents, compared to previous studies. Additionally, our findings showed that children and adolescents with higher BMI, WC, or %BF at baseline were more likely to develop hypertension throughout the three years, highlighting the importance of pediatric health care to avoid future harm.
